# Development of a clinically useful multi-segment kinetic foot model

**DOI:** 10.1186/s13047-023-00686-0

**Published:** 2023-11-29

**Authors:** Songlin Zhu, Thomas Jenkyn

**Affiliations:** 1 Wolf Orthopaedic Biomechanics Laboratory, School of Kinesiology, Faculty of Health Sciences, Western University, London, Ontario N6A 5B9 Canada; 2 Wolf Orthopaedic Biomechanics Laboratory, Department of Mechanical and Materials Engineering, Faculty of Engineering, Western University, London, Ontario N6A 5B9 Canada

## Abstract

**Background:**

Traditionally, gait analysis studies record the foot as a single rigid segment, leaving movements and loads within the foot undetected. In addition, very few data of multi-segment foot kinetics have been represented in the literature due to measurement and equipment limitations. As a result, this study aims to develop a novel multi-segment kinetic foot model that is clinically feasible and enables both kinematic and kinetic analysis of large patient groups.

**Results:**

Outcome measurements include rotation angles of intersegmental dorsi/plantar flexion, inversion/eversion, and internal/external rotation, joint moments, joint powers and the medial longitudinal arch (MLA) height/length ratio. Repeatability of joint motions was calculated using coefficients of multiple correlation. Most joint motions measured by this foot model showed strong within-subject reliability (R > 0.7) in healthy adults. Outcome measures were in agreement with other multi-segment foot models found in the biomechanics literature.

**Conclusions:**

This novel multi-segment foot model is able to quantify intersegmental foot kinematics and kinetics and can be a useful tool for research and assessments on clinical populations.

## Introduction

Clinical gait analysis with optical motion capture traditionally records the foot as a single rigid segment that articulates with the lower leg. However, this setup is unable to capture clinically important motions that occur within the foot, such as the rise and fall of the medial longitudinal arch (MLA) and the dorsiflexion of the hallux. To address this, several multi-segment foot models that enable measurement of within-foot kinematics with optical motion capture have been proposed and tested on different clinical populations [2, 9, 16, 18, 23].

Multi-segment foot models divide the foot into separate foot segments and track the relative motion between each adjacent segment. However, there is no consensus in the literature as to how the foot segments should be defined. Although each foot model defines a different number of segments or defines the segments in different ways, there are two segments that most foot models tend to have in common: the hindfoot and the forefoot. The rest of the foot is then further divided into segments differently depending on the foot model and its intended application [2, 16, 23].

Each foot segment requires at least three markers be placed on it to track its six degree-of-freedom position and orientation. This increases the overall number of markers that must be placed on the foot from the three that are usual for a single-segment foot model. This added complexity of the marker set increases the risk of errors in marker placement in a clinical setting [2, 23]. In many patients and test subjects, the foot is simply not big enough to accommodate all the markers needed. As a result, the number of additional markers required for construction of foot segments has to be limited for a multi-segment foot model to be clinically feasible. In contrast, defining too few foot segments simply will not add much useful information compared to a single-foot model. For instance, many foot models omit segments for the midfoot and hallux [9, 11, 18]. Both of these segments are functionally important as the midfoot forms the arches of the foot and the hallux plays a significant role in push off at the end of the stance phase. The importance of including the midfoot and the hallux along with the hindfoot and forefoot is increasingly being identified, hence clinically important within-foot motions are not missed [17, 21].

While many multi-segment kinematic foot models have been proposed, there are very few foot models that measure intersegmental foot kinetics [5, 8, 11]. It has been shown that treating the foot as a single kinetic segment overestimates ankle joint kinetics in both healthy and pathological gait [13]. However, the few existing multi-segment kinetic foot models have several drawbacks. Some require visual targeting by the test subject that alters natural gait [5]. Others require additional equipment for pedobarographic data that is not available in most clinical settings [13, 23, 24]. Many define segments with limited clinical relevance or oversimplify joints within foot [1, 4, 23–25]. To date, these drawbacks have limited the clinical use of multi-segment kinetic foot models either as a research tool or a clinical assessment tool.

The objective of this study is to develop a novel multi-segment kinetic foot model that is clinically feasible and enables the simultaneous recording of kinematic and kinetic analysis of large patient groups. The DuPont foot model, first proposed by Henley and colleagues [15], was used as the foundation and modified for this study. Although the original model only included three foot segments (hindfoot, forefoot, hallux), its sparse configuration of individual auto-reflective markers makes it relatively easy to modify foot segmentation by adding markers. For this study, a midfoot segment was added to the original model with three additional markers on the foot.

The secondary objective of this study is to introduce a methodology by which the intersegmental kinetics can be qualified as this has not been attempted with the DuPont model and most of other foot models. In this study, intersegmental foot kinetics will be measured using a force plate and an optical motion capture system that has already been used by traditional gait analysis.

## Methods

Testing took place in the Wolf Orthopaedic Biomechanics Laboratory at the Fowler Kennedy Sport Medicine Clinic in London, Ontario, Canada. The lab is equipped with a twelve-camera optical motion capture system (Kestrels and Raptor cameras, Motion Analysis Corporation, Santa Rosa, CA, USA). Cameras were placed symmetrically around a ten-meter walkway and sampled at 60 frames per second. Kinematic data were filtered by a low-pass Butterworth filter at a cut-off frequency of 6 Hz. Post-processing was done with Cortex 7.0 software (Motion Analysis Corporation). A floor-mounted force plate (AMTI Biomechanics, Watertown, MA, USA) measured the ground reaction forces (GRFs) and was sampled at 1200 Hz.

The healthy study population was ten young adults (3 males, 7 females; average age 22 ± 2 years old; average height 169.2 ± 8.5 cm; average weight 64 ± 11 kg; average BMI 22.3 ± 2.6 kg/m^2^) who all volunteered for the study. None of the participants had any musculoskeletal disorders, previous musculoskeletal injuries, or on-going symptoms. None had any obvious lower limb malalignment. This study was approved by our institution’s Research Ethics Board and written and verbal informed consent was obtained from every participant.

**Fig. 1:**
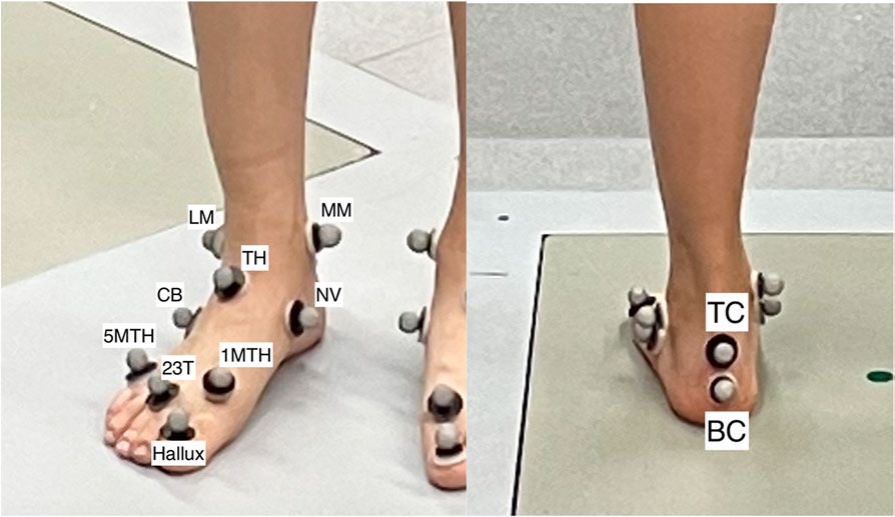
Modified DuPont foot model markers placements. LM: lateral malleolus, MM: medial malleolus, TC: top calcaneus, BC: bottom calcaneus, TH: talar head, NV: navicular bone, CB: cuboid, 1MTH: first metatarsal head, 5MTH: fifth metatarsal head, 23 T: between the heads of second and third metatarsals and the Hallux

Individual auto-reflective markers were attached to the skin using double-sided tape. The marker configuration was the modified Helen Hayes [7] on the body and the modified DuPont foot model marker set [15], which has eleven markers on the foot. These are at the center of the proximal posterior calcaneus (top calcaneus), the center of the distal posterior calcaneus (bottom calcaneus), medial and lateral malleoli, navicular, talar head, cuboid, heads of the first and fifth metatarsals, between the heads of second and third metatarsals, and the hallux nail bed (Fig. 1). The talar head marker was added to the original DuPont model to create a midfoot segment. This model has four functional foot segments in total: hindfoot, midfoot, forefoot, hallux. Segment-fixed coordinate systems are defined for each foot segment.

**Fig. 2:**
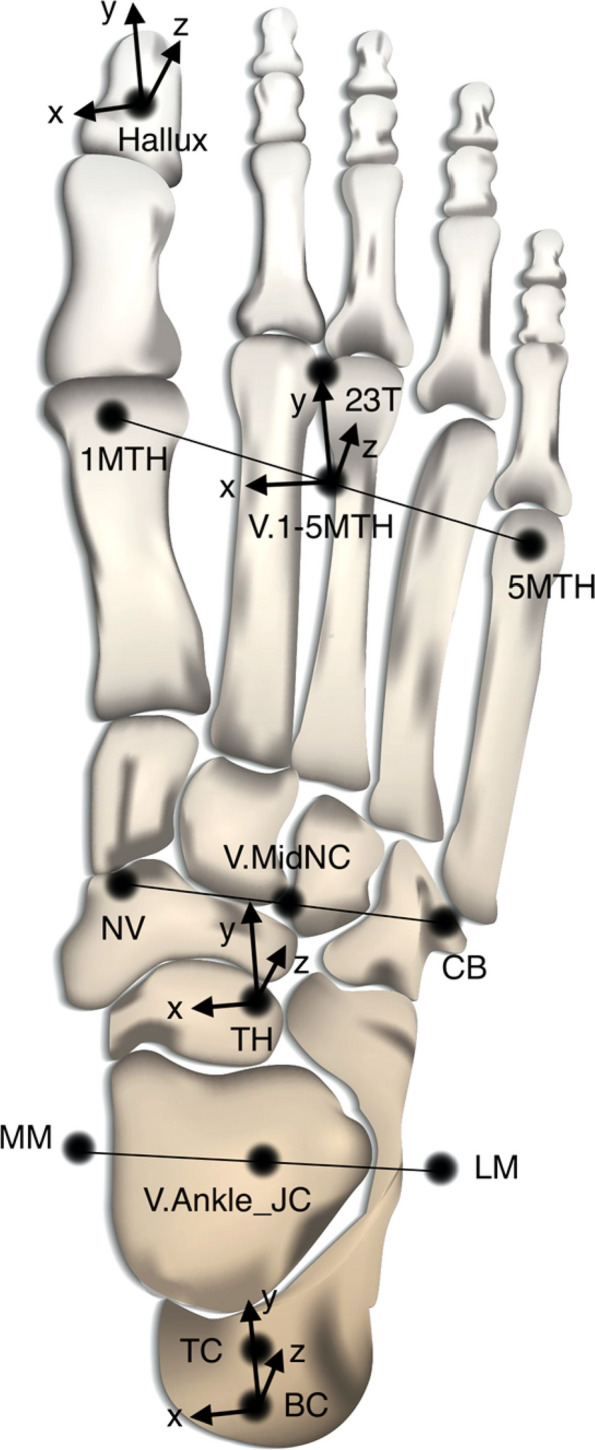
Axis directions for each foot segment as defined by the Cortex 7.0 Skeleton Builder

The markers that define each of the segment-fixed reference frames of the lower body segments are listed in Table 1. For each joint, the distal segment moved relative to the proximal (parent) segment. The Cortex 7.0 software Skeleton Builders function uses three markers, namely Origin marker, Long Axis marker, and Plane marker, to define a body segment. Origin marker represents the proximal end of a segment (e.g. a proximal joint center or a proximal end point). Long Axis marker represents the distal end of a segment (e.g. a distal joint center or a distal end point). The Long Axis is defined from Origin marker to Long Axis marker. This is labeled the z-axis, and the length of a segment was determined by the distance between Origin marker and Long Axis marker. Plane marker is the third marker used to define the y–z plane together with Origin marker and Long Axis marker. The x-axis is then created perpendicular to the y–z plane via the right-hand rule. The y-axis is created as orthogonal to the x- and z-axes. For all foot segments, the x-axis was medio-lateral pointing to the left, the y-axis was vertical pointing upwards, and the z-axis pointed anteriorly. The axes were each rotated via the RX, RY and RZ offsets (parameters in degree to orient the local coordinate system) listed in Table 1 so all foot segment axes align in the same directions. The axis directions for each foot segment are shown in Fig. 2.

**Table 1:** Lower body segment definitions in the Cortex software

Segment	Parent	Origin Marker	Long Axis Marker	Plane Marker	RX Offset	RY Offset	RZ Offset
pelvis	Global	V_Pelvis_Origin	V_Mid_Hip	V.Sacral	0	0	180
R.Thigh	pelvis	V_R.Hip_JC	V_R.Knee_JC	R.Knee	0	0	-90
L.Thigh	pelvis	V_L.Hip_JC	V_L.Knee_JC	L.Knee	0	0	90
R.Shank	R.Thigh	V_R.Knee_JC	V_R.Ankle_JC	R.Ankle	0	0	-90
L.Shank	L.Thigh	V_L.Knee_JC	V_L.Ankle_JC	L.Ankle	0	0	90
R.Hindfoot	R.Shank	R.BC	V_R.Ankle_JC	R.TC	0	0	0
L.Hindfoot	L.Shank	L.BC	V_L.Ankle_JC	L.TC	0	0	0
R.Midfoot	R.Hindfoot	V_R.Ankle_JC	R.TH	V_R.MidNC	0	0	180
L.Midfoot	L.Hindfoot	V_L.Ankle_JC	L.TH	V_L.MidNC	0	0	180
R.Forefoot	R.Midfoot	V_R.MidNC	R.1-5MTH	R.NV	0	0	90
L.Forefoot	L.Midfoot	V_L.MidNC	L.1-5MTH	L.NV	0	0	-90
R.Hallux	R.Forefoot	R.1st MTH	R.Hallux	R.23 T	0	0	-90
L.Hallux	L.Forefoot	L.1st MTH	L.Hallux	L.23 T	0	0	90

Global: the global coordinate system; *V_* Virtual markers, *JC* Joint center, *BC* Bottom calcaneus, *TC* Top calcaneus, *TH* Talar head, *MidNC* The midpoint between navicular and cuboid, *1-5MTH* The midpoint between the 1^st^ and 5^th^ metatarsal heads, *NV* Navicular, *23 T* between the 2^nd^ and 3^rd^ metatarsal heads

**Fig. 3:**
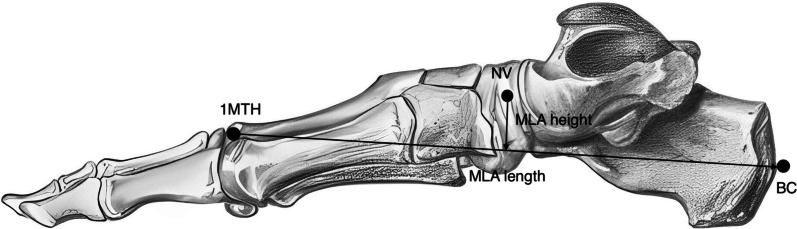
MLA length and height calculation

Intersegmental motions were measured as the distal segment relative to the proximal segment. Intersegmental rotations were calculated by the x–z-y Cardan angle sequence, with ‘x’ being dorsiflexion/plantar flexion, ‘z’ being inversion/eversion and ‘y’ being internal rotation/external rotation. The medial longitudinal arch (MLA) height/length ratio was also calculated. The length of MLA was the distance between the 1MTH marker and BC marker. The height of MLA was the magnitude of the perpendicular vector from the NV marker to the 1MTH-BC vector (MLA length). Figure 3 indicates how MLA length and height are calculated in our model.

Multi-segment foot kinetics were calculated using the measured multi-segment foot kinematics, GRFs and anthropometric data via an inverse dynamics analysis [28]. Unlike Bruening et al.’s method [4] and Dixon et al.’s method [11], only one force plate was used (rather than two) to measure ground reaction forces. Intersegment joint moments and powers were measured starting at heel-strike on the force plate for all foot segments.

The mass of each foot segment was apportioned according to its volume as reported in Drillis et al.'s paper [12], assuming homogeneous density of the foot. Definitions of foot segments in our model were slightly different from those in the Drillis et al.’s paper [12]. The “base of foot” in Drillis et al. [12] was considered as the hindfoot in our model. The “middle foot” was the forefoot plus the midfoot in our model. The volume of the hindfoot in our model was assumed to be half of the overall volume of “base of foot” and “middle foot” reported by Drillis et al. [12]. The forefoot and the midfoot in our model together equally took the other half of the overall volume of “base of foot” and “middle foot”. The hallux in our study was equivalent to the “five toes” in Drillis et al.’s paper [12]. The radii of gyrations of foot segments were determined according to De Leva [10]. The centers of mass were assumed to be halfway along the segment long axis.

All kinematic and kinetic curves were normalized from 0 to 100% of the gait cycle (101 data points) by linear interpolation of data points and averaged over three walking trials for all ten participants. Within-subject coefficients of multiple correlation (CMCs) (denoted R) were calculated over the three walking trials of each of the ten participants to test the reliability of the current multi-segment foot model. Test–retest CMCs were assessed on two participants due to limited lab access during COVID-19. The multi-segment kinematic foot model was compared with the original DuPont model [22, 26], Leardini’s model [20] and Jenkyn and Nicol’s model [16]. The multi-segment kinetic foot model was compared with Bruening et al.’s model [4] and Saraswat et al.’s model [24].

## Results

**Fig. 4:**
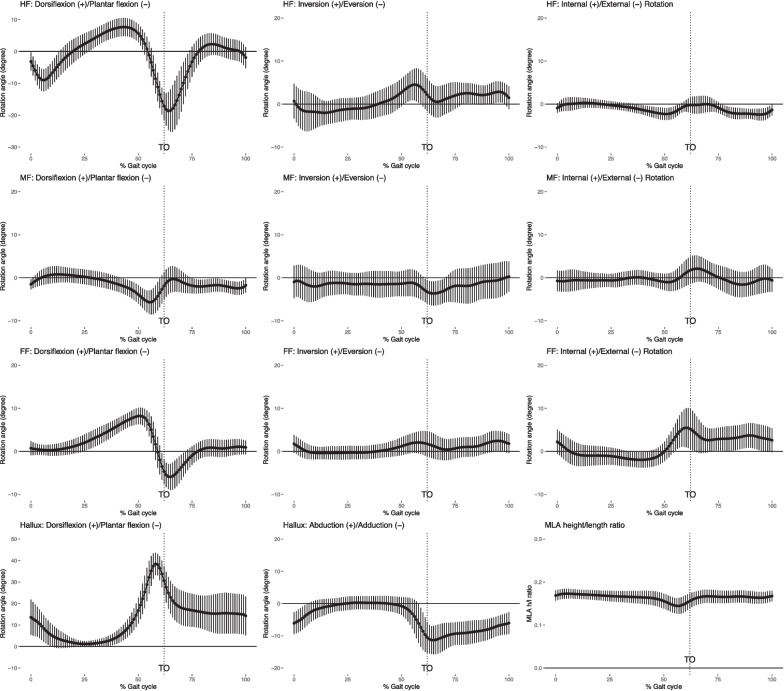
Intersegmental rotation angles for foot segments in a gait cycle. Plots for the hindfoot (HF), midfoot (MF), forefoot (FF), and hallux are shown in rows from top to bottom in order. Plots for movements in the sagittal plane, frontal plane and transverse plane are shown in column from left to right in order. Dorsiflexion, inversion or abduction and internal rotation are plotted as positive. The bottom right graph shows the MLA height-to-length ratio in a gait cycle. The average curve is plotted with one positive and one negative standard deviation. Toe off (TO) is indicated using a vertical dotted line

**Fig. 5:**
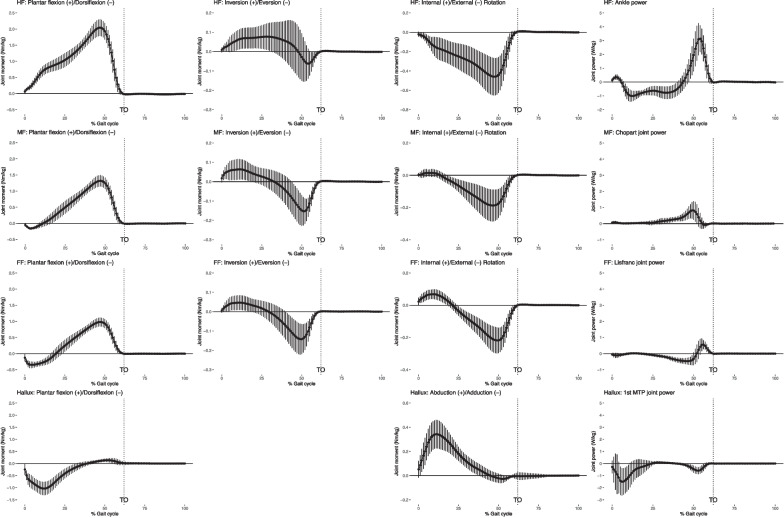
Intersegmental joint moments and powers for foot segments in a gait cycle. Plots for the hindfoot (HF), midfoot (MF), forefoot (FF), and hallux are shown in rows from top to bottom in order. Plots for joint moments in the sagittal plane, frontal plane and transverse plane are shown in column from left to right in order. Joint powers are shown in the right most column for the ankle (HF with respect to the lower leg, top row), Chopart joint (MF to HF, second row), Lisfranc joint (FF to MF, third row) and the first metatarsal-phalangeal joint (Hallux to FF, bottom row). Plantarflex, invert or abduct and internally rotate are plotted as positive. The average curve is plotted with one positive and one negative standard deviation. Toe off (TO) is indicated using a vertical dotted line

Average walking speed of ten participants was 1.22 ± 0.17 m/s and the average stride length was 1.31 ± 0.15 m. Kinematic outcome measures (intersegmental rotation angles and MLA height/length ratio) for a normalized gait cycle are shown in Fig. 4. Kinetic outcome measures (intersegmental joint moments and powers) for a normalized gait cycle are shown in Fig. 5. Joint moments and powers are normalized by the body mass in kilograms. The stance phase takes on average 60% of a gait cycle. The swing phase accounts for the rest 40% of gait cycle after toe off (TO).

For foot kinematics, hindfoot and forefoot showed similar trends but different ROMs for all three motions, while midfoot showed a reversed trend in dorsiflexion/plantar flexion. MLA height/length ratio showed a typical pattern of descending during the stance phase, reaching its nadir slightly before TO then bouncing back at TO and into the swing phase. Hallux had a large ROM (close to 40 degrees) in the sagittal plane. It remained neutral for most of the stance phase and adducted pushing off the ground and during the swing phase. For foot kinetics, hindfoot, midfoot and forefoot showed similar trends in joint moments in all three motions, while hallux demonstrated a different profile in dorsiflexion/plantar flexion and abduction/adduction moments. Ankle generated the higher power and the other foot joints had smaller powers.

Two participants were tested for a second time on a different day for test–retest reliability. Within-subject coefficients of multiple correlation (CMCs) and test–retest CMCs for intersegmental rotation angles, joint moments and powers are shown in Tables 2, 3 and 4 respectively. For the rotation angle curves, all intersegmental angles showed strong within-subject and test–retest reliability (R ≥ 0.7), however hindfoot inversion/eversion, midfoot internal/external rotation, and midfoot inversion/eversion showed moderate test–retest reliability (0.3 ≤ R < 0.7). Midfoot inversion/eversion showed moderate within-subject reliability (R = 0.66). Joint moments for all foot segments in all three planes and all joint powers showed strong within-subject and test–retest reliability (R ≥ 0.8).

**Table 2:** Within-subject coefficients of multiple correlation (CMCs) and test–retest CMCs for intersegmental rotation angles with standard deviation (SD)

		**Rotation angle curves**			
**Segment**	**Joint motion**	**Within-subject CMC**	**SD**	**Test–retest CMC**	**SD**
**Hindfoot**	Dorsiflexion / Plantar flexion	0.96	0.02	0.95	0.05
	Internal / External rotation	0.89	0.06	0.75	0.16
	Inversion / Eversion	0.75	0.13	**0.64**^a^	0.05
**Midfoot**	Dorsiflexion / Plantar flexion	0.90	0.04	0.80	0.12
	Internal / External rotation	0.79	0.08	**0.34**^a^	0.01
	Inversion / Eversion	**0.66**^a^	0.15	**0.49**^a^	0.34
**Forefoot**	Dorsiflexion /Plantar flexion	0.94	0.03	0.94	0.06
	Internal / External rotation	0.93	0.03	0.91	0.05
	Inversion / Eversion	0.84	0.04	0.88	0.02
**Hallux**	Dorsiflexion / Plantar flexion	0.96	0.03	0.96	0.03
	Abduction / adduction	0.91	0.05	0.75	0.20
	MLA height/length ratio	0.90	0.05	0.71	0.18

^a^denotes 0.3 ≤ R < 0.7 or moderate reliability

**Table 3:** Within-subject CMC and test–retest CMC and standard deviation for intersegmental foot joint moments

Segment	Joint motion	Joint moment curves			
		**Within-subject CMC**	**SD**	**Test–retest CMC**	**SD**
**Hindfoot**	Dorsiflexion / Plantar flexion	0.99	0.01	0.99	0.00
	Internal / External rotation	0.97	0.01	0.99	0.01
	Inversion / Eversion	0.91	0.06	0.92	0.09
**Midfoot**	Dorsiflexion / Plantar flexion	0.98	0.01	0.99	0.01
	Internal / External rotation	0.94	0.03	0.94	0.03
	Inversion / Eversion	0.93	0.04	0.96	0.01
**Forefoot**	Dorsiflexion /Plantar flexion	0.98	0.01	0.98	0.02
	Internal / External rotation	0.98	0.01	0.95	0.06
	Inversion / Eversion	0.87	0.10	0.79	0.23
**Hallux**	Dorsiflexion / Plantar flexion	0.94	0.05	0.97	0.02
	Abduction / adduction	0.92	0.06	0.96	0.03

**Table 4:** Within-subject CMC and test–retest CMC and standard deviation for intersegmental foot joint powers

Joint	Joint power curves			
	**Within-subject CMC**	**SD**	**Test–retest CMC**	**SD**
Ankle (Hindfoot to lower leg)	0.94	0.04	0.95	0.05
Chopart joint (Midfoot to Hindfoot)	0.83	0.08	0.96	0.02
Lisfranc joint (Forefoot to Midfoot)	0.85	0.09	0.92	0.08
1^st^ MTP (Hallux to Forefoot)	0.80	0.11	0.85	0.11

## Discussion

A novel multi-segment foot has been developed in this study based on a modified DuPont foot model [15]. One extra marker is added to the dorsum of foot at the talar head to create a midfoot segment. There are four foot segments in total: hindfoot (calcaneus), midfoot (cuneiforms, navicular, cuboid), forefoot (five metatarsals), and hallux. Intersegmental rotation angles were measured relative to the proximal foot segment using the x–z-y Cardan angle sequence and described in all three anatomical planes (dorsi/plantar flexion, inversion/eversion, internal/external rotation). Results showed that twenty-six out of twenty-seven outcome measures demonstrated strong within-subject reliability (R > 0.7).

In order to compare the current model with other multi-segment foot models in the literature, ranges of motion (ROMs) of each foot segment in the current model are listed in Table 5 for comparison with models by Lee et al. [22], Leardini et al. [20] and Jenkyn et al. [16]. Due to different foot segmentation methods and the use of x–z-y Cardan angle convention instead of the joint coordinate system by Grood and Suntay [14], our model did show differences in ROMs compared to the other three models. Our model’s forefoot ROMs, hallux ROMs and hindfoot dorsiflexion/plantar flexion ROM are comparable to Lee et al.’s model [22], but its ROMs of internal/external rotation and inversion/eversion are smaller. This is likely because the addition of midfoot now reveals motions that are inclusively measured in Lee et al.’s definition of hindfoot. Our model’s midfoot ROMs are comparable to Leardini et al.’s model [20].

**Table 5:** Comparison of intersegmental ranges of motion (ROMs) in the current model with other foot models in the literature [16, 22]

**Segment**	**Joint motion**	**Current model (*n* = 10)**	**Lee et al. [**22**] (female,** ***n* = 50)**	Leardini et al. [20]** (*n* = 9)**	Jenkyn and Nicol [16]** (*n* = 12)**
		**ROM (Max, Min)**	**ROM (Max, Min)**	**ROM (Max, Min)**	**ROM (Max, Min)**
**Hindfoot**	Dorsiflexion ( +) /Plantar flexion (-)	26.30 (7.65, -18.65)	25.5 (14.0, -11.5)	12.0 (4.7, -7.3)	15 (5, -10)
	Internal ( +) / External rotation (-)	2.71 (0.28, -2.44)	12.8 (11.3, -1.5)	10.7 (4.5, -6.2)	
	Inversion ( +) / Eversion (-)	6.56 (4.53, -2.03)	13.2 (9.8, -3.4)	9.5 (2.2, -7.3)	10 (5, -5)
**Midfoot**	Dorsiflexion ( +) /Plantar flexion (-)	6.46 (0.77, -5.69)		8.0 (2.5, -5.5)	
	Internal ( +) / External rotation (-)	3.71 (2.10, -1.61)		2.8 (2.1, -0.7)	8 (6, -2)
	Inversion ( +) / Eversion (-)	3.93 (0.27, -3.66)		4.4 (3.0, -1.4)	11 (8, -3)
**Forefoot**	Dorsiflexion ( +) /Plantar flexion (-)	14.17 (8.23, -5.95)	14.8 (3.5, -11.2)	11.5 (12.6, 1.1)	
	Internal ( +) / External rotation (-)	7.48 (5.50, -1,98)	10.5 (-0.5, -11.0)	6.9 (3.7, -3.2)	
	Inversion ( +) / Eversion (-)	2.82 (2.44, -0.39)	8.7 (11.8, 3.1)	14.3 (13.3, -1.0)	12 (15, 3)
**Hallux**	Dorsiflexion ( +) /Plantar flexion (-)	37.29 (38.47, 1.18)	40.3 (30.8, -9.5)	25.6 (26.8, 1.2)	
	Abduction ( +) / adduction (-)	11.63 (0.27, -11.37)	10.4 (0.9, -11.3)	15.7 (1.0, -14.7)	
	MLA height/length ratio	0.03 (0.17, 0.14)	0.06 (0.25, 0.19)		0.4 (1.3, 0.9)

**Fig. 6:**
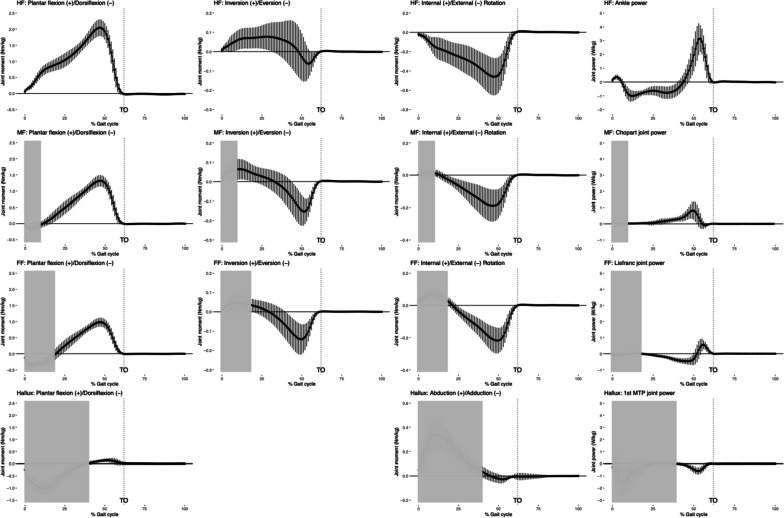
Intersegmental joint moments and powers for foot segments in a gait cycle using the “CPcross” method

The multi-segment foot kinetic measures generally agree with Bruening et al. [4] and Saraswat et al. [24] in terms of joint patterns and magnitudes. However, the current model measured a large dorsiflexion moment, abduction moment and energy absorption (power valley) at the 1st metatarsophalangeal (MTP) joint in early stance phase that were absent in Bruening et al. [4] and Saraswat et al. [24]. It is speculated that the methods adopted to measure GRFs may have led to differences in hallux kinetic profiles. Bruening et al.’s study [4] used two adjacent force plates to partition the foot at the mid-tarsal joint and 1st MTP joint and measure GRFs applied to adjacent foot segments separately. Saraswat et al.’s study [24] combined GRF data with plantar pressure data to identify when and for how long a foot segment was loaded and used the ratio of segmental vertical forces to partition shear forces accordingly. Both methods omitted the inertial effects of foot segments during walking since the whole foot has a relatively small inertial effects compared to the whole body [4, 24]. When only one force plate is used to measure GRFs and the inertial effects of foot segments are disregarded, a calculation method “CPcross” has been proposed in the literature. It quantifies GRFs only when the center of pressure crossed anterior to the joint [6, 27]. If we apply “CPcross” to our model, GRFs sequentially crossed the proximal joint of the hindfoot, midfoot, forefoot and hallux at around 0%, 12%, 23%, and 41% of a gait cycle respectively, and thus joint moments and powers for each foot segment will be calculated and plotted starting at the corresponding time point when GRFs crossed its proximal joint. Figure 6 shows the new multi-segment foot kinetic profiles of our model using “CPcross”. It is based on Fig. 5, with plots before GRFs crossed the proximal joint of each foot segment ignored (covered by grey opaque blocks). The hallux generated a peak plantar flexion moment of 0.14Nm/kg, a peak adduction moment of 0.03Nm/kg, and the minimal power was -0.59W/kg. The new hallux (1st MTP joint) moment and power curves, in particular, are highly similar in patterns and magnitude compared to Bruening et al.’s [4] and Saraswat et al.’s [24] models.

The reduced repeatability of midfoot motions (0.3 ≤ R < 0.7) is likely due to the smaller ROM of the midfoot relative to its adjacent segments, so that small errors in marker placement may have a larger effect on the calculated orientation of the segment-fixed axes. Additionally, the midfoot lacks a typical movement pattern during a gait cycle in our sample likely because it is supposed to be stable in healthy individuals during walking, especially in the frontal and transverse planes. The midfoot is worth tracking clinically since reduced or increased mobility of the midfoot during walking can suggest pathological gait when compared to healthy controls [3, 19, 24].

There were some limitations with this study. First, the masses and inertial properties for the foot segments were assumed based on anthropometric data from De Leva et al. [10] and Drillis et al. [12] rather than experimentally determined. In the absence of better data, these assumptions are considered reasonable and close to the actual properties. Small deviations likely have little effect on the kinetic output since the GRF applied to the foot is much larger in comparison to the small masses of foot segments. Second, this study used only one force plate without foot partitioning when measuring GRFs in this study. As a result, GRFs could be oversimplified when more than one segment is on the ground (e.g., during midstance). The breakdown of GRFs applied to each foot segment upon contact with the ground cannot be detected due to limited spatial and temporal resolutions of the force plate. Moreover, the direction of joint moments could be influenced by the relative position between the overall center of pressure on the force plate and the centers of mass of segments. However, the current model quantified the inertial effects of foot segments that have been neglected in previous multi-segment kinetic foot models. Only two participants were tested for test–retest reliability. Although gait patterns of healthy individuals tend to be more stable and predictable compared to individuals with pathologies, results generated from such a small sample size are prone to error and bias. Despite the small sample size, twenty-four out of twenty-seven outcome measures showed R > 0.7 for test–retest comparisons. A larger sample size is needed in future studies for test–retest reliability of our foot model.

The current multi-segment kinematic and kinetic foot model is feasible for use in a clinical setting for several reasons. The model requires only eleven markers on the foot. The markers used are individual spheres and small in size, so even on small feet there is sufficient room for placement. The model defines four foot segments, including a midfoot, so that clinically important motions within foot can be captured. The model does not require any gait-altering movement protocols or extra equipment. Moreover, the majority of kinematic and kinetic calculations are done with existing motion capture software (Cortex 7.0). This is convenient for researchers and clinicians that lack knowledge in creating special coding.

In conclusion, this study supports the within-subject reliability and validity of this novel multi-segment kinematic and kinetic foot model. This model was able to quantify intersegmental foot kinetics, including the joint moments and powers at the midfoot with the current model provides novel data to the field of foot biomechanics. It can be a clinically useful tool for research and assessments on clinical populations, help us better understand foot/ankle pathologies, and potentially inform treatments like exercise and orthoses.

## Data Availability

The datasets used and/or analysed during the current study are available from the corresponding author on reasonable request.

## Abbreviations

BMI: Body mass index
ROM: Range of motion
CMC: Coefficient of multiple correlation
GRF: Ground reaction force
MLA: Medio-longitudinal arch
1MTH: First metatarsal head
5MTH: Fifth metatarsal head
23 T: Between heads of second and third metatarsals and the hallux
LM: Lateral malleolus
MM: Medial malleolus
TC: Top calcaneus
BC: Bottom calcaneus
TH: Talar head
NV: Navicular bon
CB: Cuboid
MidNC: Midpoint between navicular and cuboid
1-5MTH: Midpoint between 1 and 5MTH
MTP: Metatarsophalangeal
TO: Toe-off
